# Prevalence of excessive screen time and associated factors in adolescents

**DOI:** 10.1016/j.rpped.2015.04.001

**Published:** 2015

**Authors:** Joana Marcela Sales de Lucena, Luanna Alexandra Cheng, Thaísa Leite Mafaldo Cavalcante, Vanessa Araújo da Silva, José Cazuza de Farias

**Affiliations:** a Programa Associado de Pós-graduação em Educação Física, Universidade Federal da Paraíba (UPE/UFPB), João Pessoa, PB, Brazil; b Centro de Ciências da Saúde, Universidade Federal da Paraíba (UFPB), João Pessoa, PB, Brazil; c Grupo de Estudos e Pesquisas em Epidemiologia da Atividade Física - GEPEAF, João Pessoa, PB, Brazil

**Keywords:** Sedentary behavior, Motor activity, Obesity

## Abstract

**Objective:**

To determine the prevalence of excessive screen time and to analyze associated factors among adolescents.

**Methods:**

This was a cross-sectional school-based epidemiological study with 2874 high school adolescents with age 14-19 years (57.8% female) from public and private schools in the city of João Pessoa, PB, Northeast Brazil. Excessive screen time was defined as watching television and playing video games or using the computer for more than 2 h/day. The associated factors analyzed were: sociodemographic (gender, age, economic class, and skin color), physical activity and nutritional status of adolescents.

**Results:**

The prevalence of excessive screen time was 79.5% (95%CI 78.1-81.1) and it was higher in males (84.3%) compared to females (76.1%; *p*<0.001). In multivariate analysis, adolescent males, those aged 14-15 year old and the highest economic class had higher chances of exposure to excessive screen time. The level of physical activity and nutritional status of adolescents were not associated with excessive screen time.

**Conclusions:**

The prevalence of excessive screen time was high and varied according to sociodemographic characteristics of adolescents. It is necessary to develop interventions to reduce the excessive screen time among adolescents, particularly in subgroups with higher exposure.

## Introduction

Sedentary behaviors are low energy expenditure activities (≤1.5 metabolic equivalent - MET), usually performed in a sitting or reclining position, including activities such as watching television, using the computer, sitting at school, on the bus, in a car, at work, talking with friends, among other similar activities.^1^ The measure of the daily time that adolescents spend watching television, playing videogames and using the computer, called screen time, is one of the most often used methods to assess sedentary behavior in studies with adolescents.^2^

It is recommended that children and adolescents dedicate a maximum of 2 h a day to screen time.^3^ The report from the Health Behavior in School-Age Children (HBSC),^4^carried out with adolescents aged 11, 13 and 15 years from 41 countries in Europe and in North America, disclosed that 56-65% of them spent 2 h or more per day watching television. Data from the National Adolescent School-based Health Survey (PeNSE),^5^ carried out with ninth-grade students from public and private elementary schools in all Brazilian capitals and the Federal District, showed that 78% of the students reported watching television for 2 h or more a day. A systematic review of studies with Brazilian adolescents showed that in 60% of the studies analyzed, the prevalence of excessive screen time was over 50%.^6^

Studies on excessive screen time among Brazilian adolescents have been almost always performed regarding time spent watching television, involving samples at young age groups and using different cut points.^7^^,^ 8 Moreover, in most cases, they were carried out in the south and southeast regions, reducing generalization of findings,^6^ due to the fact that these regions are economically more developed, which promotes greater access to electronic devices (computer and Internet access), which would stimulate the adoption of sedentary behaviors.^9^ The National Sample Survey of Households (PNAD, 2013),^9^ showed that in the south and southeast regions the proportion of households with a computer is 52.9% and 56.5%, respectively, with Internet access being present in 44.6% and 50.2% of households, respectively. In the Northeast, this prevalence is lower, with 29.4% of households with a computer and 25.3% with Internet access.

The high prevalence of adolescents exposed to excessive screen time is a matter of concern because of its association with several health problems, such as overweight and obesity, alterations in blood glucose and cholesterol, poor school performance, decreased social interaction and lower levels of physical activity.^10^^-^ 12 It is also noteworthy the fact that excessive screen time in adolescence can persist into adulthood.^13^However, the associations between excessive screen time measures and overweight/obesity in adolescents are still contradictory.^14^^,^ 15 These results may be related to the cut points used to define excessive screen time, as well as the employed measures (objective vs. subjective), the age groups of adolescents and the several study designs (cross-sectional vs. longitudinal).^14^^,^ 15

Regarding the possible influences of excessive screen time on the physical activity levels of adolescents, the data are still insufficient to confirm the hypothesis that this behavior substitutes the time spent practicing moderate to vigorous physical activity.^11^ When identified, the association between excessive screen time and physical activity levels of adolescents shows to be of low magnitude and varies according to the measure of physical activity used.^1^^,^ 2

Information on the prevalence of adolescents in northeastern Brazil with excessive exposure to sedentary behaviors, particularly screen time, their distribution in sociodemographic strata, as well as screen time association with excess body weight and physical activity practice level are important knowledge gaps that need to be filled. Thus, this study aimed to determine the prevalence of excessive screen time and to analyze its association with sociodemographic factors, physical activity level and nutritional status in adolescents from northeast Brazilian city.

## Method

This study is part of a research project carried out in 2009, entitled: "Physical activity level and associated factors among high school adolescents in João Pessoa, city, - PB: an ecological approach." The target population consisted of high school students, aged 14-19 years, from public and private schools João Pessoa city, state of Paraiba, Brazil. To determine sample size, a prevalence of 50% of 300 min or more of moderate to vigorous physical activity per week was used, with a maximum acceptable error of three percentage points, 95% confidence interval, design effect (deff) equal to two and addition of 30% to compensate losses and refusals, resulting in a sample of 2686 adolescents.

Sample selection was performed by two-stage cluster. In the first stage, 30 high schools were systematically selected, distributed proportionally by size (number of enrolled students), by type (public and private) and regions of the city (north, south, east, west). In the second, 135 classes were randomly selected, proportionally distributed by shift (daytime and nighttime) and high school grade (1st, 2nd and 3rd year of high school).

Data collection occurred from May to September 2009 and was carried out by a team of six Physical Education undergraduate students who had been previously trained and submitted to a pilot study. All information was collected through a previously tested questionnaire, filled out by students in class and during regular class time, following instructions provided by the collection team.

Sociodemographic variables analyzed in this study were gender, age in years (determined from the difference between date of birth and the data collection date, and categorized as 14-15, 16-17 and 18-19 year) and socioeconomic class, determined by the methodology of the Brazilian Association of Research Enterprises - ABEP,^16^ which considers the presence of material goods and salaried employees in the residence, as well as the educational level of the head of the household. The students were grouped in socioeconomic classes A1, A2, B1, B2, C1, C2, D and E, and later re-categorized as: class A/B (high), C (middle) and D/E (low).

Nutritional status was estimated by body mass index (BMI = body weight [kg]/height [m^2^]), based on self-reported measures of body mass (kg) and height (cm). The criteria suggested by Cole et al. 17 were used to classify adolescents as "without excess body weight" (low weight + normal weight) and "with excess body weight" (overweight + obesity).

The sociodemographic variable measurement reproducibility was high, with kappa (*κ*) values ≥0.89 *p*<0,01. BMI showed an intraclass correlation coefficient (ICC=0.95) and *κ*value=0.84 for nutritional status (without vs. with excess body weight).

Excessive screen time was assessed based on the measurement of mean daily time (hours/minutes) spent in front of television and playing videogames and/or using the computer, on weekdays and the weekend, during a typical or usual week. For the final result, the weighted mean was calculated from the following: summation of time spent in sedentary behaviors on weekdays (Monday-Friday) multiplied by five, added to time spent on the weekend (Saturday or Sunday) multiplied by two. This result was divided by seven to obtain the mean number of hours a day that the adolescents spent on screen activities. Excessive screen time was defined as spending more than 2h per day in these behaviors.^3^ Screen time measurement showed satisfactory levels of reproducibility (continuous measure [hours/day]-ICC=0.76; *p*<0,01; categorical measure [≤2 h/day vs. >2h/day] - *κ*=0.52).

Physical activity was measured using a previously validated questionnaire (reproducibility-ICC=0.88; 95%CI: 0.84-0.91; validity-Spearman correlation=0.62; *p*<0.001; *κ*=0.59).^18^ The adolescents reported the frequency (days/week) and duration (minutes/day) of moderate to vigorous physical activities practiced in the week before data collection for at least 10 min, considering a list of 24 activities, with the possibility to add two more activities. Physical activity level was determined by adding the product of time by the frequency of practice in each activity, resulting in a score in minutes per week. Adolescents who performed 300 min or more of physical activity per week were classified as "physically active", and others, as "physically inactive".

Initially, descriptive statistics procedures (frequency distribution, mean, standard deviation and 95% confidence interval - 95%CI) were used. The chi-square test was used to compare the proportion of adolescents showing excessive screen time as a function of sociodemographic variable categories (gender, age, skin color, socioeconomic class), level of physical activity ("physically active" and "physically inactive") and nutritional status ("without excess body weight" and "with excess body weight").

Logistic regression was used to assess the association between excessive screen time and sociodemographic variables, level of physical activity and nutritional status. The model had excessive screen time as the dependent variable (≤2h/day=0 and >2h/day=1) and as independent variables: gender (female=1, male=2), age (14-15=3; 16-17=2, and 18-19 years =1), skin color (White =1 and non-White =2), socioeconomic class (class A/B=3, C=2, D/E=1), level of physical activity (physically active=1; physically inactive=2) and nutritional status (without excess body weight=1; with excess body weight=2). In the adjusted analysis, all the independent variables were included in the model and those with *p*-value <0.20 remained. The backward method was applied to select variables in the multivariate model. The Hosmer-Lemeshow test was used to assess the model goodness-of-fit.

The study was approved by the Institutional Review Board of Universidade Federal da Paraíba (Protocol 0062/2009). All adolescents younger than 18 years received authorization from parents or guardians to participate, and those older than 18 years signed the informed consent form to participate in this study.

## Results

Initially, a total of 3477 students were selected to participate in the study, but 70 were not allowed by their parents or guardians or refused to participate, and 187 were not available on at least three visits of the research team. Of the 3220 adolescents who answered the questionnaire, 346 were excluded (231 were <14 or>19 years of age, 105 did not inform the age, 5 had left several unanswered questions and 5 had some type of physical or mental limitation). The final sample included 2874 adolescents, of which 57.8% were females, mean age of 16.5±1.2 years; 54.2% were of middle to low socioeconomic class (C/D/E), 50.2% were physically active and 13.2% had excess body weight (Table 1).

**Table 1:** Sociodemographic characteristics, physical activity and nutritional status of high school adolescents from public and private schools of João Pessoa, northeastern Brazil, in 2009.

Variables	*n*	%
*Gender*		
Female	1653	57.8
Male	1206	42.2

*Age range (years)*		
14–15	1128	10.7
16–17	1438	50.0
18–19	308	39.3

*Skin color*		
White	930	32.5
Non-white	1929	67.5

*Socioeconomic class*		
A and B (high)	1161	45.8
C (middle)	1167	46.1
D and E (low)	205	8.1

*Physical activity level*		
Physically active	1444	50.2
Physically inactive	1430	49.8

*Nutritional status*		
No excess body weight	2321	86.8
Excess body weight	353	13.2

The prevalence of excessive screen time was 79.5% (95%CI: 78.1-81.1), being higher in males (*p*<0.001), in youngest (14-15 years; *p*<0.001), those of higher socioeconomic class (*p*<0.001) and among the physically active ones (*p*=0.002). There were no statistically significant differences between adolescents with or without excess body weight (Table 2).

**Table 2:** Prevalence of excessive screen time and associated factors in high school adolescents from public and private schools of João Pessoa, northeastern Brazil, 2009.

Variables	Excessive screen time^a^			
	Prevalence (%)	95%CI	*p*
*Total*	79.5		

*Gender*			<0.001
Female	76.1	73.9–78.1	
Male	84.3	82.1–86.2	

*Skin color*			0.075
White	81.5	78.8–88.9	
Non-white	78.6	76.7–80.4	

*Age range (years)*			<0.001
14–15	82.1	79.8–84.3	
16–17	79.3	77.1–81.3	
18–19	71.1	66.6–75.9	

*Socioeconomic status*			<0.001
A and B (high)	84.6	82.4–86.5	
C (middle)	78.6	76.0–80.8	
D and E (low)	60.8	53.7–67.3	

*Physical activity level*			0.002
Physically active	81.8	79.7–83.7	
Physically inactive	77.2	74.9–79.3	

*Nutritional status*			0.139
No excess body weight	79.0	77.3–80.6	
Excess body weight	82.4	78.0–86.1	

a Watching TV, using the computer and playing videogames for more than 2 h/day.

At the crude analysis (Table 3), excessive screen time was associated with gender, age, socioeconomic class and level of physical activity of the adolescents. In the adjusted analysis, the level of physical activity lost statistical significance, while the other variables remained associated with excessive screen time. Male adolescents, aged 14-15 years and from higher socioeconomic classes (classes A/B) had respectively 49% (OR=1.49; 95%CI: 1.21-1.84), 46% (OR=1.46; 95%CI: 1.07-2.00) and 224% (OR=3.24; 95%CI: 2.32-4.52) higher chance of exposure to excessive screen time, compared to females, older adolescents (18-19 years) and those from the lower socioeconomic classes (C/D/E). The result of the Hosmer-Lemeshow test (*χ* ^2^ =8.26; *p*=0.22) showed that the constructed model was well adjusted to the data.

**Table 3:** Crude and adjusted analyses for the association between excessive screen time and associated factors in adolescents from public and private schools of João Pessoa, northeastern Brazil, 2009.

Variables	Excessive screen time^a^			
	Crude OR (95%CI)	*p*	Adjusted OR^b^ (95%CI)	*p*
*Gender*		<0.001		<0.001
Female	1		1	
Male	1.68 (1.39–2.04)		1.49 (1.21–1.84)	

*Skin color*		0.075		
White	1		–	
Non-white	0.83 (0.68–1.02)		–	

*Age range (years)*		<0.001		<0.001
14–15	1.87 (1.40–2.51)		1.46 (1.07–2.00)	
16–17	1.56 (1.18–2.06)		1.31 (1.30–2.51)	
18–19	1		1	

*Socioeconomic status*		<0.001		<0.001
A and B (high)	3.54 (2.56–4.91)		3.24 (2.32–4.52)	
C (middle)	2.36 (1.72–3.24)		2.27 (1.64–3.13)	
D and E (low)	1		1	

*Physical activity level*		0.002		
Physically active	1		–	
Physically inactive	1.33 (1.11–1.60)		–	

*Nutritional status*		0.140		
No excess body weight	1		–	
Excess body weight	1.25 (0.93–1.67)		–	

a Watching TV, using the computer and playing videogames for more than 2h/day.

b Analysis adjusted by sociodemographic variables, physical activity level and nutritional status.– Data showed no significant association after adjustment (*p*>0.05).

## Discussion

In this study, the proportion of adolescents who showed excessive screen time was high and varied according to their sociodemographic characteristics. Higher chances of excessive screen time were found in adolescent males, younger ones and those from the higher socioeconomic classes. Contrary to what has been speculated in the literature, excessive screen time was not associated with excess body weight and low levels of physical activity among adolescents.

This study showed that approximately 8 of 10 adolescents spent more than 2 h a day on screen activities (television, computer and videogames). Similar results have also been identified in other national^8^^,^ 19 and international studies.^12^^,^ 14 ^,^ 20 High prevalence of excessive screen time, often observed in adolescents, may result from changes in society over the past two to three decades, such as economic growth that allowed families, especially middle-low income ones, to major access to television, computer, greater use of the Internet in leisure time (e.g. interaction in social networks) and reduction of public spaces for physical activities, combined with the observed lack of safety in large urban centers. A study carried out between 2001 and 2011 with adolescents aged 15-19 years in the state of Santa Catarina observed a reduction in the prevalence of TV time and increased computer/videogame use. These changes were attributed to economic changes in Brazil, easier access to computers (Internet cafes, shopping malls, public places) and access to electronic media in general.^21^

In the present study, male adolescents were approximately 49% more likely to have excessive screen time, reinforcing findings of previous studies.^7^^,^ 8 ^,^ 19 One reason for this high prevalence among male adolescents is mainly caused by excess use of videogames and computer.^20^ Studies that identified a higher prevalence of excessive time spent in sedentary behaviors in females measured, in addition to screen activities, the time spent talking on the phone, listening to music, doing homework, writing or talking.^22^^,^ 23 Another factor that may explain these differences is the cut point used to characterize excessive screen time.^14^ A systematic review^6^ demonstrated that the prevalence resulting from the use of the cut point of 2 h or more of television time was higher in males, while that derived from the cut point of 4 h or more was higher in females.^6^ Cultural issues may also help to understand these differences. Female adolescents are more encouraged to stay at home for longer periods and are raised with greater care and to devote themselves to studies and household chores.^24^ This would lead to greater prevalence of sedentary behaviors.

Increased exposure of adolescents to excessive screen time may be related to the adolescents’ phase of "cultural definition". Up to 14-15 years old, most of the activities are divided between school, household chores and friends.^24^ At this phase, adolescents follow rules set by their parents, who generally limit their activities to the home environment.^24^ Therefore, these factors contribute to the increased use of time using electronic devices, such as computer, videogames and watching television. These activities, in addition to being among the forms of social interaction of adolescents (through social networks) are also adopted by their groups of friends, which reinforces the adoption of these behaviors through the social influence of friends.^22^ The greater involvement with excessive screen time at this age range may indicate less social interaction and less restriction from parents about the time spent using computers, playing videogames and watching television.^13^ Another explanation is that, possibly, older adolescents (around 16 years) would be involved and would be encouraged by their parents to become apprentices, take courses and study for in the university. Thus, the time dedicated to studying and other social activities would limit screen time or, logically, would reduce the interest in these activities.

The higher exposure of adolescents from the higher socioeconomic classes (A/B) to excessive screen time observed in this study may be associated with a greater chance of these adolescents having videogames and computer at home, especially with Internet access. Data from the National Adolescent School-based Health Survey^5^ showed that among students enrolled in the 9th grade of Elementary school, 95.5% of private school students had a computer (desktop, netbook, laptop), compared to 59.8% of students from public schools. This is supported by the results that were found including only the time spent watching television. It was observed that the adolescents from classes D/E were more likely to watch television for more than 2 h, when compared to their peers from classes A/B (OR=1.78; 95%CI: 1.28-2.49 - data not shown in table). These results are similar to those found in other national studies that used the measure of television time as the sedentary behavior outcome.^7^^,^ 8 Coombs et al. 25 observed that the association between screen time and socioeconomic class varied according to the type of sedentary behavior: adolescents from lower socioeconomic classes spent more time watching TV and less time on other sedentary behaviors, such as doing homework, drawing, using the computer or playing videogames.

It should be noted that even though this study used excessive screen time as sedentary behavior indicator, the proportion of households in the state of Paraíba in 2009 that had a computer was small (19%) when compared to households with a TV (95.5%).^26^ Thus, it is possible that the differences between the socioeconomic classes regarding the type of sedentary behavior assessed in the study result from the higher access of adolescents from higher socioeconomic classes to the computer and video games, while those belonging to the lower classes may have higher access to television. Although television is still the most popular means of entertainment and leisure in all social strata and represents the largest share of screen time, future researches should include other types of electronic devices, such as the use of cell phones and/or tablets, as well as Internet access, which are less frequent in the poorer socioeconomic classes.

No significant association was identified between excess body weight and excessive screen time. Studies that observed a positive association between screen time and excess body weight, in most cases, only measured the time spent watching television.^27^^,^ 28 A systematic review of cross-sectional studies showed a positive association between screen time and excess body weight, but these associations were not identified in studies using objective measures of sedentary behavior.^14^ Corroborating these findings, a review of longitudinal studies published in 2011 concluded that there was evidence supporting the association between screen time and excess body weight in adolescents.^29^ These results demonstrate that the association of screen time and excess weight can vary according to the study design and used measure of sedentary behavior.

One explanation for the association between excessive screen time and excess body weight in adolescents may be related to increased consumption of unhealthy foods, such as soft drinks, snacks and candy in front of the television and exposure to fast food ads, considering that television remains the primary means of communication for advertisements.^30^ These factors influence food intake and diet quality, resulting in a positive energy balance and, consequently, increase in body weight.^11^^,^ 27

The lack of a significant association between excessive screen time and physical activity levels identified in this study is similar to that observed in other studies with adolescents.^7^^,^ 28 Physical activity and sedentary behaviors are distinct constructs with "determinants", associated factors and specific health implications.^2^^,^ 12 In this context, an individual can be physically active (i.e., meet the moderate to vigorous physical activity recommendations) and still have excessive sedentary behavior time.^1^ Systematic reviews have demonstrated that the association between sedentary behaviors and physical activity, when significant, showed very low magnitude,^14^^,^ 31 and that interventions to increase physical activity practice levels had no significant effects and, when they did, they showed low magnitude to reduce the time observed in sedentary behaviors.^32^^,^ 33

Martins et al. 28 found that correlated adverse factors for physical activity practice were not associated with sedentary behavior in adolescents. These results suggest that excessive exposure to sedentary behaviors does not necessarily result from adverse conditions for physical activity practice (lower perception of self-efficacy, less social support and environments for physical activity practice).

This study has some limitations. The fact that self-reported measures of weight and height were used is one of them, due to the possibility that the measures were underestimated, particularly among adolescents with body weight. However, self-reported measures have been widely used in studies of large population groups, and the results have shown to be valid.^34^Another limitation was the fact that data were not collected on the adolescents’ food consumption. Unhealthy eating habits are associated with sedentary behavior, as well as with excess body weight in this population.^11^^,^ 30 The strengths of this study were: the use of a representative sample of the adolescent population aged 14-19 years from public and private schools in the city of João Pessoa, state of Paraíba, Brazil, and a power to detect statistically significant odds ratios equal to or higher than 1.20 and prevalence of outcome in the exposed group ranging from 20% to 85%. The measure of sedentary behavior involved different activities, such as watching television, using the computer and playing videogames, and not only the time spent in front of the TV. The other strength of this study was use a pre-tested questionnaire with satisfactory levels of reproducibility, applied by a previously trained team.

The prevalence of excessive screen time was high, and male adolescents, the youngest and those of a higher socioeconomic class were the ones more exposed to this outcome. There was no significant association between screen time, nutritional status and physical activity levels of the adolescents. Longitudinal studies are needed, using objective and subjective measures to verify the association between sedentary behavior with nutritional status, eating habits and physical activity levels in adolescents.
