# Supplementary material for: Prevalence of excessive screen time and associated factors in adolescents
Source: Rev Paul Pediatr. 2015 Oct-Dec;33(4):407–14. doi: 10.1016/j.rpped.2015.04.001 (PMC4685560; doi:10.1016/j.rpped.2015.04.001)
Supplement: Translation

# Translation: Prevalência de tempo excessivo de tela e fatores associados em adolescentes

**Authors:** Joana Marcela Sales de Lucena, Luanna Alexandra Cheng, Thaísa Leite Mafaldo Cavalcante, Vanessa Araújo da Silva, José Cazuza de Farias

**Affiliations:** a Programa Associado de Pós-graduação em Educação Física, Universidade Federal da Paraíba (UPE/UFPB), João Pessoa, PB, Brasil; b Centro de Ciências da Saúde, Universidade Federal da Paraíba (UFPB), João Pessoa, PB, Brasil; c Grupo de Estudos e Pesquisas em Epidemiologia da Atividade Física - GEPEAF, João Pessoa, PB, Brasil

**Keywords:** Comportamento sedentário, Atividade motora, Obesidade

## Abstract

**Objetivo:**

Determinar a prevalência do tempo excessivo de tela e analisar fatores associados em adolescentes.

**Métodos:**

Trata-se de um estudo epidemiológico transversal, de base escolar, com 2.874 adolescentes de 14 a 19 anos de idade (57,8% do sexo feminino), do ensino médio das redes pública e privada no município de João Pessoa, PB. O tempo excessivo de tela foi definido como assistir televisão, usar o computador e jogar videogames por mais de duas horas por dia. Os fatores associados analisados foram: sociodemográficos (sexo, idade, classe econômica, cor da pele), prática de atividade física e estado nutricional do adolescente.

**Resultados:**

A prevalência de tempo excessivo de tela foi de 79,5% (IC95%: 78,1-81,1) e mais elevada no sexo masculino (84,3%) comparado com o feminino (76,1%; *p*<0,001). Na análise multivariada, verificou-se que os adolescentes do sexo masculino, os de 14 a 15 anos idade e os que pertenciam às classes econômicas mais altas apresentaram maiores chances de exposição ao tempo excessivo de tela. O nível de atividade física e o estado nutricional dos adolescentes não se associaram ao tempo excessivo de tela.

**Conclusões:**

A prevalência do tempo excessivo de tela foi elevada e variou com as características sociodemográficas dos adolescentes. Faz-se necessário desenvolver intervenções para reduzir o tempo excessivo de tela entre os adolescentes, particularmente nos subgrupos com maior exposição.

## Introdução

Os comportamentos sedentários são atividades de baixo gasto energético (≤1,5 equivalente metabólico - MET), geralmente realizadas na posição sentada ou reclinada, incluindo atividades como assistir à televisão, usar o computador, ficar sentado na escola, no ônibus, carro, trabalho, conversar com os amigos, dentre outras atividades similares.^1^ A medida do tempo que os adolescentes passam por dia assistindo à televisão, jogando videogame e usando o computador, denominada de tempo de tela (do inglês *screen time*), é uma das formas mais usadas para operacionalizar o comportamento sedentário em estudos com adolescentes.^2^

Recomenda-se que as crianças e os adolescentes limitem a, no máximo, duas horas por dia o tempo dedicado às atividades de tela.^3^ O relatório do *Health Behaviour in School-Age Children* (HBSC),^4^ realizado com adolescentes de 11, 13 e 15 anos de idade de 41 países da Europa e a América do Norte, revelou que 56% a 65% desses jovens passavam duas horas ou mais por dia assistindo à televisão. Dados da Pesquisa Nacional de Saúde do Escolar (PeNSE),^5^ com escolares do nono ano do ensino fundamental de escolas públicas e privadas de todas as capitais brasileiras e do Distrito Federal, demonstraram que 78% dos escolares relataram assistir a duas horas ou mais de televisão por dia. Revisão sistemática de estudos com adolescentes brasileiros mostrou que, em 60% dos estudos analisados, a prevalência de tempo excessivo de tela foi superior a 50%.^6^

Os estudos sobre tempo excessivo de tela em adolescentes brasileiros foram realizados, quase que na sua totalidade, com medida de tempo assistindo à televisão, envolveram amostras com faixa etária reduzida e usaram diferentes pontos de corte.^7^^,^ 8 Além disso, em sua maioria, foram realizados nas regiões Sul e Sudeste, o que limita a generalização dos achados,^6^ devido ao fato de essas regiões serem economicamente mais desenvolvidas, o que favorece um maior acesso aos dispositivos eletrônicos (computador e acesso à Internet) que estimulariam a adoção de comportamentos sedentários.^9^ A Pesquisa Nacional por Amostra em Domicílios (PNAD, 2013),^9^ demonstrou que nas regiões Sul e Sudeste a proporção de domicílios que tinham computador foi de 52,9% e 56,5% os que tinham acesso à Internet estavam á presente em 44,6% e 50,2%; respectivamente. Na região Nordeste, essa prevalência foi mais baixa, 29,4% dos domicílios com computador e 25,3% com acesso à Internet.

A elevada prevalência de adolescentes expostos a tempo excessivo de tela causa preocupação devido a sua associação com diversos problemas de saúde, como excesso de peso corporal e obesidade, alterações na glicose e colesterol sanguíneos, baixo rendimento escolar e diminuição do convívio social e menores níveis de atividade física.^10^^-^ 12 Destaca-se ainda o fato de que o tempo excessivo de tela na adolescência pode ser transferidos à idade adulta.^13^Entretanto, as associações entre medidas de tempo excessivo de tela e sobrepeso/obesidade em adolescentes ainda são conflitantes.^14^^,^ 15Esses resultados podem estar relacionados aos pontos de corte usados para definir tempo excessivo de tela, bem como as medidas empregadas (objetivas *vs*. subjetivas), às faixas etárias dos adolescentes analisadas e os desenhos dos estudos (transversais *vs*. longitudinais).^14^^,^ 15

Em relação às possíveis influências do tempo excessivo de tela sobre os níveis de atividade física dos adolescentes, os dados ainda são insuficientes para confirmar a hipótese de que esse comportamento substitui o tempo de prática de atividades físicas moderadas a vigorosas.^11^ Quando a associação entre o tempo excessivo de tela e o nível de atividade física dos adolescentes é identificada, tem sido de baixa magnitude e variado com a medida de atividade física usada.^1^^,^ 2

As informações sobre a prevalência de adolescentes da região Nordeste do Brasil com exposição excessiva aos comportamentos sedentários, particularmente para o tempo de tela, sua distribuição nos estratos sociodemográficos, bem como sua associação com excesso de peso corporal e nível de prática de atividade física, são lacunas de conhecimento importantes que precisam ser preenchidas. Sendo assim, este estudo determinou a prevalência de tempo excessivo de tela e analisou sua associação com fatores sociodemográficos, nível de atividade física e estado nutricional em adolescentes de uma cidade do Nordeste do Brasil.

## Método

Este estudo está inserido em um projeto de pesquisa maior, realizado em 2009, intitulado "Nível de atividade física e fatores associados em adolescentes do ensino médio na cidade de João Pessoa - PB: uma abordagem ecológica". A população alvo foi composta por escolares do ensino médio, de 14 a 19 anos de idade, das redes pública e privada de ensino no município de João Pessoa, PB. Para determinação do tamanho da amostra foi adotada uma prevalência de 50% de 300 minutos ou mais de atividade física moderada a vigorosa por semana, erro máximo aceitável de três pontos percentuais, intervalo de confiança de 95%, efeito de desenho (*deff*) igual a dois e acréscimo de 30% para compensar perdas e recusas, o que resultou em uma amostra de 2.686 adolescentes.

A seleção da amostra foi feita por conglomerado em dois estágios. No primeiro, foram selecionadas sistematicamente 30 escolas de ensino médio, distribuídas proporcionalmente por tamanho (número de alunos matriculados), e tipo de escola (públicas e privadas) e regiões do município (norte, sul, leste, oeste). No segundo, foram selecionadas aleatoriamente 135 turmas, distribuídas proporcionalmente por turno (diurno e noturno) e série de ensino médio (1ª, 2ª e 3ª séries).

A coleta de dados ocorreu de maio a setembro de 2009 e foi feita por uma equipe de seis estudantes do curso de educação física, previamente treinados e submetidos a um estudo piloto. Todas as informações foram coletadas por questionário previamente testado, preenchido pelos escolares na sala de aula e no horário regular de aula, de acordo com as instruções fornecidas pela equipe de coleta.

As variáveis sociodemográficas analisadas neste estudo foram: sexo, idade em anos completos (determinada a partir da diferença entre a data de nascimento e a data de coleta de dados e categorizada em: 14-15, 16-17, 18-19 anos de idade) e classe econômica, determinada pela Metodologia da Associação Brasileira de Empresas de Pesquisa (ABEP),^16^ que considera a presença de bens materiais e de empregados mensalistas na residência, além da escolaridade do chefe de família. Os estudantes foram agrupados nas classes econômicas A1, A2, B1, B2, C1, C2, D e E, posteriormente reagrupadas em classes A/B (alta), C (média) e D/E (baixa).

O estado nutricional foi estimado pelo índice de massa corporal (IMC = massa corporal [kg]/estatura [m^2^]), baseado nas medidas autorreferidas de massa corporal (kg) e estatura (cm). Os critérios sugeridos por Cole et al.^17^ foram usados para classificar os adolescentes em: "sem excesso de peso corporal" (baixo peso + peso normal) e "com excesso de peso corporal" (sobrepeso + obesidade).

A reprodutibilidade das medidas das variáveis sociodemográficas foram elevadas, com valores de kappa ≥0,89 *p*<0,01. O IMC apresentou coeficiente de correlação intraclasse (e valor de κ=0,84 para classificação do estado nutricional (sem *vs*. com CCI=0,95) excesso de peso corporal).

O tempo excessivo de tela foi operacionalizado a partir da medida do tempo médio diário (horas/minutos) despendido assistindo televisão, jogando videogames e usando o computador, nos dias de semana e do fim de semana, durante uma semana típica ou habitual. Para o resultado final, foi calculada a média ponderada a partir do seguinte procedimento: somatório do tempo despendido nos comportamentos sedentários em dias de semana (segunda a sexta) multiplicado por cinco, somado ao tempo dos dias de fim de semana (sábado ou domingo) multiplicado por dois. Esse resultado foi dividido por sete para se obter o número médio de horas por dia que os adolescentes passavam em atividades de tela. O tempo excessivo de tela foi definido como passar mais de duas horas por dia nesses comportamentos.^3^ A medida do tempo de tela apresentou níveis satisfatórios de reprodutibilidade (medida contínua [horas/dia] - CCI=0,76 *p*<0,01; medida categórica [≤2 horas/dia *vs.* >2 horas/dia] - κ=0,52).

A atividade física foi mensurada por questionário previamente validado (reprodutibilidade - CCI=0,88 *p*<0,01; validade - correlação de Spearman =0,62; *p*<0,001; κ=0,59).^18^ Os adolescentes informaram a frequência (dias/semana) e a duração (minutos/dia) das atividades físicas de intensidade moderada a vigorosa praticadas na semana anterior à coleta de dados por pelo menos 10 minutos, considerando uma lista com 24 atividades, com a possibilidade de o adolescente adicionar mais duas atividades. Determinou-se o nível de atividade física somando o produto do tempo pela frequência de prática em cada atividade, o que resultou num escore em minutos por semana. Os adolescentes que praticaram 300 minutos ou mais de atividade física por semana foram classificados como "fisicamente ativos" e os demais como "fisicamente inativos".

Inicialmente, foram adotados os procedimentos de estatística descritiva (distribuição de frequências, média, desvio padrão e intervalo de confiança de IC95%). O teste do qui-quadrado foi usado para comparar a proporção de adolescentes em tempo excessivo de tela em função das categorias das variáveis sociodemográficas (sexo, idade, cor da pele, classe econômica), nível de atividade física ("fisicamente ativo" e "fisicamente inativo") e estado nutricional ("sem excesso de peso corporal" e "com excesso de peso corporal").

A regressão logística binária foi usada para avaliar a associação entre o tempo excessivo de tela e as variáveis sociodemográficas, nível de atividade física e estado nutricional. O modelo teve como variável dependente o tempo excessivo de tela (≤2 horas/dia=0 e >2 horas/dia=1) e como variáveis independentes sexo (feminino=1, masculino=2), idade (14-15=3; 16-17=2 e 18-19 anos=1), corda pele (branca=1 e não branca=2), classe econômica(classes A/B=3; C=2; D/E=1), nível de atividade física (fisicamente ativo=1; fisicamente inativo=2) e estado nutricional (sem excesso de peso corporal=1; com excesso de peso corporal=2). Na análise ajustada, todas as variáveis independentes foram incluídas no modelo e permaneceram aquelas com valor de *p*<0,20. Foi aplicado o método *backward* para seleção das variáveis no modelo múltiplo. O teste de Hosmer-Lemeshow foi usado para avaliar a qualidade do ajuste do modelo.

O estudo foi aprovado pelo Comitê de Ética em Pesquisa com Seres Humanos do Centro de Ciências da Saúde da Universidade Federal da Paraíba - Protocolo n° 0062/2009. Todos os adolescentes menores de 18 anos de idade receberam autorização dos pais ou responsáveis e aqueles com mais de 18 anos assinaram o termo de consentimento livre e esclarecido para participar deste estudo.

## Resultados

Inicialmente foram selecionados 3.477 escolares, 70 não foram autorizados pelos pais ou responsáveis ou se recusaram a participar e 187 não foram encontrados em pelo menos três visitas da equipe de coleta de dados. Dos 3.220 adolescentes que responderam ao questionário, 346 foram excluídos (231 tinham <14 ou >19 anos de idade, 105 não informaram a idade, cinco deixaram várias questões sem resposta e cinco tinham alguma limitação de ordem física ou mental). A amostra final incluiu 2.874 adolescentes, 57,8% do sexo feminino, com média de 16,5±1,2 anos; 54,2% de classe econômica média a baixa (C/D/E), 50,2% eram fisicamente ativos e 13,2% tinham excesso de peso corporal (Tabela 1).

**Tabela 1:** Características sociodemográficas, nível de atividade física e estado nutricional dos adolescentes do ensino médio das redes pública e privada de João Pessoa, Nordeste do Brasil, 2009

Variáveis	n	%
*Sexo*		
Feminino	1.653	57,8
Masculino	1.206	42,2

*Faixa etária (anos)*		
14‐15	1.128	10,7
16‐17	1.438	50,0
18‐19	308	39,3

*Cor da pele*		
Branca	930	32,5
Não branca	1.929	67,5

*Classe econômica*		
A e B (alta)	1.161	45,8
C (média)	1.167	46,1
D e E (baixa)	205	8,1

*Nível de atividade física*		
Fisicamente ativo	1.444	50,2
Fisicamente inativo	1.430	49,8

*Estado nutricional*		
Sem excesso de peso corporal	2.321	86,8
Com excesso de peso corporal	353	13,2

A prevalência de tempo excessivo de tela foi de 79,5% (IC95%: 78,1-81,1), sendo maior no sexo masculino (*p*<0,001), nos mais jovens (14-15 anos de idade; *p*<0,001), nos de classe econômica mais alta (*p*<0,001) e nos fisicamente ativos (*p*=0,002). Não houve diferenças estatisticamente significativas entre os adolescentes com ou sem excesso de peso corporal (Tabela 2).

**Tabela 2:** Prevalência de tempo excessivo de tela e fatores associados em adolescentes do ensino médio das redes pública e privada de João Pessoa, Nordeste do Brasil, 2009

Variáveis	Tempo excessivo de tela^a^		
	Prevalência (%)	IC95%	*p*
*Total*	79,5		

*Sexo*			<0,001
Feminino	76,1	73,9‐78,1	
Masculino	84,3	82,0‐86,2	

*Cor da pele*			0,075
Branca	81,5	78,8‐88,9	
Não branca	78,6	76,7‐80,4	

*Faixa etária (anos)*			<0,001
14‐15	82,1	79,8‐84,3	
16‐17	79,3	77,1‐81,3	
18‐19	71,1	66,6‐75,9	

*Classe econômica*			<0,001
A e B (alta)	84,6	82,4‐86,5	
C (média)	78,6	76,0‐80,8	
D e E (baixa)	60,8	53,7‐67,3	

*Nível de atividade física*			0,002
Fisicamente ativo	81,8	79,7‐83,7	
Fisicamente inativo	77,2	74,9‐79,3	

*Estado nutricional*			0,139
Sem excesso de peso corporal	79,0	77,3‐80,6	
Com excesso de peso corporal	82,4	78,0‐86,1	

a Assistir televisão, usar o computador e jogar videogames por mais de duas horas/dia.

Na análise bruta (Tabela 3), o tempo excessivo de tela foi associado ao sexo, idade, classe econômica e nível de atividade física dos adolescentes. Na análise ajustada, o nível de atividade física perdeu significância estatística. As demais variáveis permaneceram associadas ao tempo excessivo de tela. Adolescentes do sexo masculino, de 14 a 15 anos de idade e os de classes econômicas mais altas (classes A/B) tiveram, respectivamente, 49% (OR=1,49; IC95%: 1,21-1,84), 46% (OR=1,46; IC95%: 1,07-2,00) e 224% (OR=3,24; IC95%: 2,32-4,52) mais chances de exposição ao tempo excessivo de tela, comparados com os adolescentes do sexo feminino, mais velhos (18-19 anos de idade) e de classes econômicas mais baixas (C/D/E). O resultado do teste de Hosmer-Lemeshow (χ^2^ =8,26; *p*=0,22) demonstrou que o modelo construído ajustou-se bem aos dados.

**Tabela 3:** Análise bruta e ajustada para associação entre tempo excessivo de tela e fatores associados em adolescentes das redes pública e privada de João Pessoa, Nordeste do Brasil, 2009

Variáveis	Tempo excessivo de tela^a^			
	OR bruta (IC95%)	*p*	OR ajustada^b^ (IC95%)	*p*
*Sexo*		<0,001		<0,001
Feminino	1		1	
Masculino	1,68 (1,39‐2,04)		1,49 (1,2‐1,84)	

*Cor da pele*		0,075		
Branca	1		–	
Não branca	0,83 (0,68‐1,02)		–	

*Faixa etária (anos)*		<0,001		<0,001
14‐15	1,87 (1,40‐2,51)		1,46 (1,07‐2,00)	
16‐17	1,56 (1,18‐2,06)		1,31 (1,30‐2,51)	
18‐19	1		1	

*Classe econômica*		<0,001		<0,001
A e B (alta)	3,54 (2,56‐4,91)		3,24 (2,32‐4,52)	
C (média)	2,36 (1,72‐3,24)		2,27 (1,64‐3,13)	
D e E (baixa)	1		1	

*Nível de atividade física*		0,002		
Fisicamente ativos	1		–	
Fisicamente inativo	1,33 (1,11‐1,60)		–	

*Estado nutricional*		0,140		
Sem excesso de peso corporal	1		–	
Com excesso de peso corporal	1,25 (0,93‐1,67)		–	

a Assistir televisão, usar o computador e jogar videogame por mais de duas horas/dia.

b Análise ajustada pelas variáveis sociodemográficas, nível de atividade física e estado nutricional.– Excluída do modelo final por apresentar valor *p*>=0,20.

## Discussão

No presente estudo verificou-se que a proporção de adolescentes em tempo excessivo de tela foi elevada e variou conforme suas características sociodemográficas. Maiores chances de permanecer em tempo excessivo de tela foram encontradas nos adolescentes do sexo masculino, nos mais jovens e de classe econômica mais alta. Ao contrário do que tem sido especulado na literatura, o tempo excessivo de tela não se associou com excesso de peso corporal e a baixos níveis de atividade física dos adolescentes.

Este estudo demonstrou que aproximadamente oito em cada dez adolescentes passavam mais de duas horas por dia em atividades de tela (televisão, computador e videogame). Resultados similares também foram identificados em outros estudos nacionais^8^^,^ 19 e internacionais.^12^^,^ 14 ^,^ 20 Prevalências elevadas de tempo excessivo de tela, frequentemente observadas em adolescentes, podem ser decorrentes das mudanças ocorridas na sociedade nas últimas duas a três décadas, como, por exemplo, o crescimento econômico que permitiu às famílias, sobretudo as de renda média-baixa, maior acesso a televisão, computador, maior uso da Internet no tempo de lazer (p. ex.; interagir nas redes sociais) e redução dos espaços públicos para a prática de atividades físicas, associada à insegurança observada nos grandes centros urbanos. Estudo conduzido entre 2001 e 2011 com adolescentes de 15 a 19 anos de idade do Estado de Santa Catarina observou uma redução da prevalência do tempo de televisão e aumento do uso de computador/videogames. Essas alterações foram atribuídas às mudanças econômicas no Brasil, facilidade de acesso a computadores (*lan houses*, shoppings, espaços públicos) e acesso as mídias eletrônicas de uma maneira geral.^21^

No presente estudo, os adolescentes do sexo masculino apresentaram aproximadamente 49% mais chances de permanecer em tempo excessivo de tela, o que reforça achados de estudos prévios.^7^^,^ 8 ^,^ 19 Um dos motivos para essa prevalência elevada entre adolescentes do sexo masculino se dá principalmente pelo uso excessivo de videogame e computador.^20^ Os estudos que identificaram maior prevalência de tempo excessivo em comportamentos sedentários no sexo feminino mensuraram, além das atividades de tela, o tempo gasto com falar ao telefone, ouvir música, fazer o dever de casa, escrever ou conversar.^22^^,^ 23Outro fator que pode explicar essas diferenças é o ponto de corte usado para caracterizar tempo excessivo de tela.^14^Estudo de revisão sistemática^6^ demonstrou que as prevalências resultantes do uso do ponto de corte de duas horas ou mais de tempo de televisão eram mais elevadas no sexo masculino, enquanto que aquelas derivadas do ponto de corte de quatro horas ou mais eram mais elevadas no feminino.^6^ Questões culturais também podem ajudar a entender essas diferenças. Adolescentes do sexo feminino são mais incentivadas a permanecer mais tempo em casa e são criadas com maiores cuidados para se dedicarem aos estudos e às tarefas do lar.^24^ Isso levaria a uma maior adoção de comportamentos.

Maior exposição dos adolescentes mais jovens ao tempo excessivo de tela pode ter relação com essa fase de "definição cultural" do adolescente. Até os 14 a 15 anos de idade, a maior parte das atividades está dividida entre a escola, as tarefas domésticas e os amigos.^24^ Nessa fase, os adolescentes seguem regras impostas pelos pais, que, em geral, limitam suas atividades ao ambiente do lar.^24^Desse modo, esses fatores contribuem para o maior tempo de uso de dispositivos eletrônicos como computador, videogame e assistir à televisão. Essas atividades, estão entre as formas de interação social dos adolescentes (por meio das redes sociais), e são também adotadas por grupos de amigos, o que reforça a adoção desses comportamentos por meio da influência social dos amigos.^22^ O maior envolvimento em tempo excessivo de tela nessa idade pode indicar menos compromissos sociais e menor restrição dos pais quanto ao tempo de uso do computador, videogame e televisão.^13^ Outra explicação é que, possivelmente, os adolescentes mais velhos (por volta dos 16 anos de idade) estariam envolvidos ou seriam estimulados pelos pais a fazer estágios, cursos e estudar para concursos ou entrada na universidade. Desse modo, o maior tempo de dedicação aos estudos e outras atividades sociais limitaria o tempo de tela ou, naturalmente, diminuiria o interesse dessas atividades.

A maior exposição dos adolescentes de classe econômica mais alta (A/B) ao tempo excessivo de tela observado no presente estudo pode estar relacionada a maior possibilidade de os adolescentes dessas classes terem videogame e computador em casa, sobretudo com acesso à Internet. Os dados da Pesquisa Nacional da Saúde do Escolar^5^ mostraram que, entre os escolares do 9° ano do ensino fundamental, 95,5% dos estudantes de escolas privadas tinham computador (de mesa, *notebook*, *laptop*), contra 59,8% de escolas públicas. Isso pode ser reforçado a partir dos resultados que foram encontrados ao considerar isoladamente o tempo assistindo à televisão. Verificou-se que os adolescentes das classes D/E tiveram maiores chances de assistir à televisão por mais de duas horas comparados com os seus pares das classes A/B (OR=1,78; IC95%: 1,280-2,49 - dados não apresentados em tabela). Esses resultados são semelhantes aos encontrados em outros estudos nacionais que usaram a medida de tempo de televisão como desfecho de comportamento sedentário.^7^^,^ 8 Coombs et al.^25^ observaram que a relação entre tempo de tela e classe econômica variava conforme o tipo de comportamento sedentário: os adolescentes de menor nível socioeconômico permaneciam mais tempo assistindo à televisão e menos tempo em outros comportamentos sedentários, como fazer o dever de casa, desenhar, usar o computador ou jogar videogame.

Deve-se ressaltar que, mesmo tendo o presente estudo utilizado o tempo excessivo de tela como indicador de comportamento sedentário, em 2009, no estado da Paraíba, a proporção de domicílios que possuía computador era pequena (19%) comparada à que possuía televisão (95,5%).^26^ Desse modo, é possível que as diferenças entre as classes econômicas em relação ao tipo de comportamento sedentário adotado sejam decorrentes do menor acesso dos adolescentes de classes de melhor condição econômica ao computador e videogame, enquanto que os que pertencem às classes menos favorecidas podem ter maior acesso à televisão. Embora a televisão ainda seja um dos meios mais populares de entretenimento e lazer em todas as camadas sociais e represente a maior parcela do tempo de tela, futuras investigações devem incluir outros tipos de dispositivos eletrônicos como o uso dos celulares e/ou *tablets* e o acesso à Internet, que são menos frequentes nas classes mais pobres da população.

Não foi identificada associação significativa entre excesso de peso corporal e tempo excessivo de tela no presente estudo. Os estudos que observaram associação positiva entre tempo de tela e excesso de peso corporal, na maioria das vezes, mediram apenas o tempo assistindo à televisão.^27^^,^ 28 Revisão sistemática de estudos transversais demonstrou uma relação positiva entre tempo de tela e o excesso de peso corporal, porém essas associações não foram identificadas nos estudos que usaram medidas objetivas de comportamentos sedentários.^14^ Corroborando esses achados, uma revisão de estudos longitudinais, publicada em 2011, concluiu que existiam evidências que sustentam a associação entre tempo de tela e excesso de peso corporal em adolescentes.^29^ Esses resultados demonstraram que a associação entre tempo de tela e excesso de peso pode variar de acordo com o delineamento do estudo e medida de comportamento sedentário usada.

Uma das explicações para a associação entre tempo excessivo de tela e excesso de peso corporal nos adolescentes pode estar relacionada ao maior consumo de alimentos não saudáveis, como refrigerantes, salgadinhos e guloseimas à frente da televisão, e pela exposição a propagandas de *fast-food*, pois a televisão ainda é o principal meio de comunicação para veiculação de propagandas.^30^ Esses fatores influenciam o consumo de alimentos e a qualidade da dieta e resultam em um balanço energético positivo e, por conseguinte, aumento do peso corporal.^11^^,^ 27

A ausência de associação significativa entre tempo excessivo de tela e nível de atividade física identificada no presente estudo é semelhante ao de outros estudos com adolescentes.^7^^,^ 28 A atividade física e os comportamentos sedentários são construtos distintos, com "determinantes", fatores correlatos e diferentes implicações para a saúde.^2^^,^ 12 Nesse contexto, uma pessoa pode ser fisicamente ativa (atender às recomendações de atividade física para a saúde) e ainda assim apresentar tempo excessivo em comportamentos sedentários.^1^ Revisões sistemáticas demonstraram que a associação entre comportamentos sedentários e atividade física, quando significativas, tinham muito baixa magnitude^14^^,^ 31 e que as intervenções para aumentar os níveis de prática de atividade física não apresentavam efeitos significativos, de baixa magnitude para reduzir o tempo em comportamentos sedentários.^32^^,^ 33

Martins et al.^28^ verificaram que fatores correlatos adversos para a prática de atividade física não estavam associados aos comportamentos sedentários em adolescentes. Esses resultados sugerem que a exposição excessiva aos comportamentos sedentários não decorre necessariamente de condições adversas para a prática de atividades físicas (menor percepção de autoeficácia, menos apoio social e ambientes prática de atividade física).

Este estudo apresenta algumas limitações. Ter usado medidas autorreferidas de peso e estatura representa uma delas, em decorrência da possibilidade de as medidas serem subestimadas, sobretudo entre adolescentes com excesso de peso corporal. No entanto, medidas autorreferidas têm sido amplamente empregadas em estudos com grandes grupos populacionais e os resultados têm se mostrado válidos.^34^ Outra limitação foi não ter coletado dados sobre o consumo alimentar dos adolescentes. Hábitos alimentares não saudáveis se associam aos comportamentos sedentários, bem como ao excesso de peso corporal nessa população.^11^^,^ 30 Os pontos fortes deste estudo foram: usar uma amostra representativa da população de adolescentes de 14 a 19 anos de idade de escolas públicas e privadas do município de João Pessoa, PB, e com poder para detectar *odds ratio* como estatisticamente significativos com valores iguais ou superiores a 1,20 e prevalência do desfecho nos expostos variando de 20% a 85%. A medida de comportamento sedentário envolveu diferentes atividades, como assistir à televisão, usar o computador e jogar videogame, e não só o tempo diante da televisão. Outro ponto forte deste estudo foi usar um questionário previamente testado, com níveis satisfatórios de reprodutibilidade, aplicado por equipe previamente treinada.

A prevalência do tempo excessivo de tela foi elevada e os adolescentes do sexo masculino, os mais jovens e os de classe econômica mais alta foram os que se mostraram mais expostos a esse desfecho. Não foi encontrada associação significativa entre tempo de tela, estado nutricional e nível de atividade física dos adolescentes. São necessários estudos longitudinais utilizando medidas objetivas e subjetivas para analisar a relação dos comportamentos sedentários com o estado nutricional, hábitos alimentares e prática de atividade física em adolescentes.
